# The influence of social constraints on the quality of life of hematopoietic stem cell transplantation survivors: The chain mediating effect of illness perceptions and the fear of cancer recurrence

**DOI:** 10.3389/fpsyg.2022.1017561

**Published:** 2022-11-25

**Authors:** Zhiying Shen, Shuangjiao Shi, Chengyuan Li, Chunhong Ruan

**Affiliations:** ^1^Department of Hematology, Third Xiangya Hospital, Central South University, Changsha, China; ^2^Clinical Nursing Safety Management Research Center of Central South University, Third Xiangya Hospital, Central South University, Changsha, China; ^3^Department of Nursing, Third Xiangya Hospital, Central South University, Changsha, China

**Keywords:** hematopoietic stem cell transplantation, survivors, social constraints, illness perceptions, fear of cancer recurrence, quality of life

## Abstract

**Objective:**

This cross-sectional correlational study aims to explore the relationship between social constraints and the quality of life of hematopoietic stem cell transplantation (HCT) survivors. Additionally, we also seek to demonstrate the chain mediating effect of illness perceptions and the fear of cancer recurrence on this relationship.

**Methods:**

Convenience sampling was employed in this study. A total of 232 HCT survivors were interviewed using the Social Constraints Scale, the Brief Illness Perception Questionnaire, the Fear of Cancer Recurrence Inventory (Short Form) and the Functional Assessment of Cancer Therapy–Bone Marrow Transplant. IBM SPSS 24.0 were used for data analyses, and PROCESS macro (Model 6) was used to examine the hypothesized chain mediation model.

**Results:**

A positive relationship between social constraints and quality of life verified the mediating effect of illness perceptions and the fear of cancer recurrence on this relationship. Social constraints affect the quality of life of HCT survivors *via* three pathways: the mediating role of illness perceptions, the mediating role of fear of cancer recurrence and the chain mediating effect of both factors.

**Conclusion:**

The chain mediating effect of illness perceptions and the fear of cancer recurrence on quality of life indicates that these two variables have important practical significance with respect to improving HCT survivors’ physical and mental health. The study thus serves as a reference for health workers to improve HCT survivors’ quality of life in the future.

## Introduction

Hematopoietic stem cell transplantation (HCT) has become a common therapeutic procedure for patients with hematological malignancies and many other life-threatening blood disorders. Although the survival rate of patients after HCT is increasing, survivors continue to be threatened by adverse events such as relapse and chronic graft-versus-host disease (c-GVHD; Pidala et al., 2011; Wang et al., 2018). Quality of life is a multidimensional concept that includes patients’ perceptions of the impact of their disease and treatment on their physical, psychological, and social functioning in daily life (Kluetz et al., 2016). Studies have shown that HCT survivors in China tend to exhibit worse quality of life than members of noncancer populations (Liang et al., 2018; Pan et al., 2021). Therefore, it is necessary for medical workers to take reasonable measures to improve the quality of life of HCT survivors.

The social-cognitive processing model indicates that an individual’s social network plays an important role in facilitating the cognitive processing of cancer-related memories, thoughts, and concerns (Lepore, 2001; Lepore and Revenson, 2007). Like psychological functioning, the various aspects of social functioning can be divided into positive and negative dimensions. Social support is generally viewed as positive (Cohen et al., 2001). In contrast, social constraint has negative connotations and is defined as “the objective social conditions and individuals’ construal of those conditions that lead individuals to refrain from or modify their disclosure of stress- and trauma-related thoughts, feelings, or concerns” (Lepore, 2001; Lepore and Revenson, 2007). In general, social support is associated with reduced pain and increase happiness, while social constraints are often associated with various forms of distress, such as anxiety, depression, fear and posttraumatic stress disorder (Rivera Rivera and Burris, 2020). Numerous researchers have demonstrated the effects of HCT on survivors’ interpersonal relationships and social networks (Sharin et al., 2020; Parisek et al., 2021). However, no study has yet investigated the influences of social constraints on the quality of life of HCT survivors. Several studies conducted to investigate cancer survivors have indicate a negative association between social constraints and quality of life (You and Lu, 2014; Cui et al., 2021; Llave and Hoyt, 2022). Therefore, this study proposed the hypothesis that social constraints negatively predict the quality of life of HCT survivors.

With respect to the relationship between social constraints and the quality of life of HCT survivors, illness perceptions are a possible mediator. The perceptions of illness is defined as a person’s thoughts about how an illness is caused, how long it will last, how it will affect his or her life, and how it can be controlled or cured (Janssen et al., 2013). A longitudinal study showed that social constraints positively predicted illness perception in cancer patients, indicating that perceived communication constraints related to social networks could lead to persistent emotional distress and negative perceptions of disease (Reng, 2021). Similarly, our previous survey of adolescents and young adults who received HCT also found that greater social constraints were associated with worse illness perceptions (Shen et al., 2022). Moreover, negative perceptions of illness largely influence the behavior and emotions exhibited by cancer survivors, leading to reduced quality of life. The results of several studies have shown that cancer patients with negative illness perceptions exhibited poorer quality of life than those who perceived their illness positively or neutrally (Fanakidou et al., 2018; Ośmiałowska et al., 2022). Thus, this study hypothesized that illness perception is a mediating variable in the relationship between social constraints and quality of life in HCT survivors.

The fear of cancer recurrence, which is defined as “fear, worry or concern relating to the possibility that cancer will come back or progress,” is considered to be a normal response to cancer diagnosis (Lebel et al., 2016). According to the conceptual model of fear of cancer recurrence proposed by Simonelli et al. (2017), social constraints negatively moderates patient’s appraisal and processing of internal (e.g., physical symptoms and side effects) or external (e.g., cancer-related media and medical follow-up) triggers, resulting in varying degrees of fear of cancer recurrence. Subsequently, the fear of recurrence affects the patient’s psychological and behavioral health outcomes. Researchers have found that HCT survivors whose fear of cancer recurrence was stronger experienced lower quality of life (Sarkar et al., 2014; Brice et al., 2020). Therefore, fear of cancer recurrence may also be an important mediator in the relationship between social constraints and quality of life.

Notably, illness perceptions and fear of cancer recurrence are not separate intermediaries. The self-regulation model of illness posits that the key factor associated with the fear of cancer recurrence is constituted by illness perceptions, emphasizing the fact that individuals respond negatively to trigger factors due to their negative cognitions regarding disease (Lee-Jones et al., 1997; Fardell et al., 2016). Further evidence has shown that individuals who viewed cancer as a chronic disease with uncontrollable negative consequences exhibited more fear of cancer recurrence than those who had positive attitudes regarding the disease (Lee-Jones et al., 1997). This position was corroborated by researchers who investigated HCT patients over a period of 1 year and found that those who experienced more control over their cancer status and had a better understanding of their disease engaged in better mental health and active health practices (Nelson et al., 2019). Therefore, we speculate that illness perceptions can influence quality of life *via* the fear of cancer recurrence.

**Figure 1:**
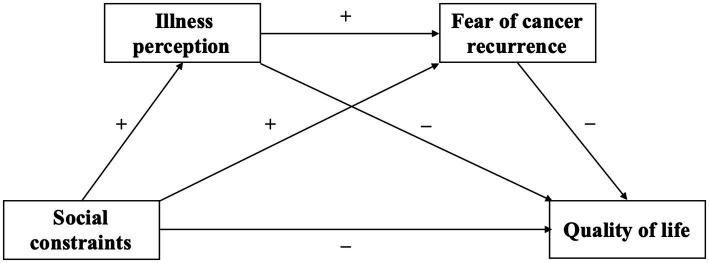
The proposed chain mediation model.

In conclusion, this study intends to explore the internal mechanism underlying the impact of social constraints on the quality of life of HCT survivors and to construct a chain mediation model of the effect of social constraints on HCT survivors’ quality of life using illness perceptions and the fear of cancer recurrence as mediating variables. Thus, according to the social-cognitive processing model, the conceptual model of fear of cancer recurrence, the self-regulation model of illness, and recent studies, we propose four hypotheses in this study (Figure 1).

*H1*: Social constraints negatively predict the quality of life of HCT survivors.

*H2*: Social constraints can indirectly predict the quality of life of HCT survivors through the intermediary role of illness perceptions.

*H3*: Social constraints can indirectly predict the quality of life of HCT survivors through the intermediary role of fear of cancer recurrence.

*H4*: Social constraints can indirectly predict the quality of life of HCT survivors through the chain mediating effect of illness perceptions and the fear of cancer recurrence.

To discuss these issues has its theoretical and practical significance. Initially, to explore the process mechanism of social constraints and the quality of life of HCT survivors in China might enrich the relevant theoretical basis. Further, knowledge about the specific role of illness perception and the fear of cancer recurrence in the relationship between social constraints and the quality of life may help medical professionals to develop effective interventions from the perspective of reducing social constraints, so as to guide HCT survivors to build up positive illness perception, reduce fear of cancer recurrence and improve quality of life.

## Materials and methods

### Participants

This cross-sectional correlational study was conducted at the Third Xiangya Hospital of Central South University in Hunan Province, China, using a convenience sampling technique. Patients were recruited from the hospital’s outpatient and HCT database. The inclusion criteria for survivors were as follows: (1) age older than 18 years; (2) ability to speak Mandarin and read Chinese questionnaires; (3) having undergone HCT between 3 months and 5 years prior to the commencement of the survey; (4) ability to use a smartphone, tablet or computer, and have access to internet; and (5) an understanding of the purpose and process of the study and provision of informed consent. Conversely, survivors were excluded if they (1) were diagnosed with other serious diseases, such as other cancers, acute myocardial infarction, cerebral hemorrhage or chronic renal failure; (2) were diagnosed with psychological or mental impairment or were receiving psychotherapy; or (3) had experienced cancer recurrence. The sample size was calculated using sample size formula for descriptive cross-sectional study N = (*u*_α_σ/δ)^2^. The sample size was estimated in due considering a 95% confidence level, and the admissible error did not exceed 3. According to the results of preliminary investigation, the mean score of the quality of life of HCT survivors was 97.32 ± 21.66, and the calculated sample size was 200. Considering the invalid rate of 20%, the final sample size was 250.

### Procedures

Data were collected from May to December 2021.The on-site investigation was conducted by two trained nurses in the HCT clinic. Each HCT survivor in the outpatient department was provided with information concerning the purpose, content, and procedures of the investigation as well as the assurance of anonymity. Upon signing an informed consent form, each survivor completed a self-administered (self-completed) questionnaire. Thereafter, all completed questionnaires were immediately collected on site and checked for missing information to ensure data integrity. In addition, we conducted an online survey, as part of which survivors were invited to complete the same questionnaire *via* a social networking site (WeChat). The survivors were screened from the hospital’s HCT database and invited to participate *via* telephone, following which they were sent a link to an online questionnaire. Survivors who agreed to participate provide verbal consent. To improve the quality of the online survey, the questionnaire could only be complete once by each IP address, and participants with a completion time of less than 5 min were excluded from further analysis. A total of 250 questionnaires were collected, 232 of which were valid, for an effective rate of 92.8%. Participants included 125 males and 107 females: 145 participants were married, 72 were unmarried, 15 were divorced or widowed; and 144 had diagnoses of acute leukemia, and 88 had diagnoses of other diseases. The types of HCT were autologous HCT for 36 participants and allogeneic HCT for 196 participants. A total of 57 participants developed c-GVHD, of which 21 (37%) were mild, 20 (35%) were moderate, and 16 (28%) were severe (according to the National Institutes of Health consensus criteria; Filipovich et al., 2005).

### Measurements

#### Social constraints

Social constraints were measured using the Social Constraints Scale (SCS; Lepore et al., 1996). This scale is a 15-item self-report measure of social responses that inhibit the expression of cancer-specific thoughts, feelings, and experiences. Each item is scored on a 4-point Likert response scale (1 = Never, 2 = Occasionally, 3 = Sometimes, 4 = Always). The range of possible total scores is from 15 to 60, with a higher score implying higher levels of social constraints. The Chinese version of the SCS was translated by You and Lu (2014), and acceptable reliability and validity were identified (Cronbach’s α coefficient = 0.91). In this study, the Cronbach’s α coefficient of the scale was 0.84.

#### Illness perceptions

Illness perceptions were measured using the Brief Illness Perception Questionnaire (BIPQ; Broadbent et al., 2006). This scale is a 9-item questionnaire that measures survivors’ cognitive and emotional representations of any illness or health condition. Answers are scored on a 10-point Likert scale, with the exception of item 9 (not included in the total score), which is an open-ended question concerning patients’ self-attributions of illness (range: 0–80). A higher total score implies a higher negative perception of illness. The Chinese version of BIPQ had been proved to have good reliability and validity in breast cancer patients (Reng, 2021). The Cronbach’s α coefficient of the scale in this study was 0.71.

#### Fear of cancer recurrence

Fear of cancer recurrence was measured using the Fear of Cancer Recurrence Inventory Short Form (FCRI-SF; Simard and Savard, 2015). This scale is a 9-item self-report questionnaire that can be used to screen and measure outcomes related to assessing the fear of cancer recurrence. Specifically, the questionnaire assesses the presence, frequency, intensity, and duration of thoughts associated with the fear of cancer recurrence. Total scores on this measure range from 0 to 36, with higher scores indicating higher levels of fear of cancer recurrence. The Chinese version of the FCRI-SF was translated by Su, and good reliability and validity (Cronbach’s α coefficient = 0.90) were identified in Chinese cancer patients (Su, 2017). The Cronbach’s α coefficient of the FCRI-SF in this study was 0.74.

#### Quality of life

The Functional Assessment of Cancer Therapy–Bone Marrow Transplant (FACT-BMT) Version 4 is a self-administered instrument designed to assess multidimensional aspects of the quality of life in HCT patients. The Chinese version of the FACT-BMT consists of the 27-item FACT-General (FACT-G) and the 10-item Bone Marrow Transplantation Subscale (BMTS; Lau et al., 2002). The FACT-G assesses four primary dimensions of quality of life, including physical well-being (7 items), social/family well-being (7 items), emotional well-being (6 items), and functional well-being (7 items). The FACT-BMTS contains 12 items, including 10 scored items. All items included in the FACT-BMT are rated on a five-point Likert scale (0 = not at all, 1 = a little bit, 2 = somewhat, 3 = quite a bit, 4 = very much). Total scores on this measure range from 0 to 148, with higher scores indicating higher levels of quality of life. The Cronbach’s α coefficients of the Chinese version of FACT-BMT was 0.92 (Lau et al., 2002). The Cronbach’s α coefficient of the scale in this study was 0.83.

## Data analysis

All data were analyzed using SPSS 24.0 software (IBM Corp., Armonk, NY, United States). Since moderate to severe chronic GVHD is the leading cause of impaired quality of life and death after HCT (Pidala et al., 2011), independent-sample *t*-test was used to compare the differences between patients with no/mild c-GVHD (no/mild c-GVHD group) and patients with moderate/severe c-GVHD (moderate/severe c-GVHD group) in social constraints, illness perceptions, the fear of cancer recurrence, and quality of life scores. Pearson correlation analysis was used to determine the correlation among social constraints, illness perceptions, the fear of cancer recurrence and quality of life. Then, to examine the hypothesized chain mediation model, we used the PROCESS macro (Model 6), with social constraints as the independent variable, the quality of life as the dependent variable, illness perceptions and the fear of cancer recurrence as the intermediate chain variables, and treating sociodemographic and clinical characteristics (gender, age, marital status, cancer diagnosis, type of HCT, and c-GVHD) as control variables for examining the chain mediating effect of illness perceptions and the fear of cancer recurrence. The moderate/severe c-GVHD group were analyzed together with the no/mild c-GVHD group due to small sample size (36 cases). For the significance test of the regression coefficient, the bootstrapping method (5,000 bootstrap samples) was used to calculate the standard error and a 95% confidence interval (CI). The effect is considered to be statistically significant if the CI does not contain zero. Furthermore, we conducted Harman’s single-factor test to determine whether common method biases affected this study. The results indicated six factors with eigenvalues greater than 1; among these factors, the largest factor explained 22.56% of the variance, i.e., less than the critical range of 40%. Therefore, no common method bias affected this study.

## Results

### Descriptive statistics and correlation analyses

Table 1 shows the descriptive statistics and correlative statistics among the variables. Social constraints were positively correlated with illness perceptions (*r* = 0.274, *p* < 0.01) and the fear of cancer recurrence (*r* = 0.311, *p* < 0.01). Furthermore, social constraints (*r* = 0.305, *p* < 0.01), illness perceptions (*r* = −0.309, *p* < 0.01), and the fear of cancer recurrence (*r* = −0.480, *p* < 0.01) were significantly negatively correlated with quality of life. Table 2 shows the results of comparisons between the no/mild c-GVHD group and the moderate/severe c-GVHD group on all measures. The score of quality of life was higher for the no/mild c-GVHD group compared with the moderate/severe c-GVHD group (*t* = 2.487, *p* = 0.014). However, there were no statistically significant differences between the two groups in scores on social constraints (*t* = 0.551, *p* = 0.291), illness perception (*t* = 0.439, *p* = 0.661) and the fear of cancer recurrence (*t* = 1.325, *p* = 0.186).

**Table 1:** Descriptive statistics and interrelations among all observed variables.

Variables	M	SD	Social constraints	Illness perception	Fear of cancer recurrence	Quality of life
Social constraints	29.10	6.89	1			
Illness perception	38.86	9.13	0.274^**^	1		
Fear of cancer recurrence	13.92	5.28	0.311^**^	0.624^**^	1	
Quality of life	95.95	20.39	−0.305^**^	−0.309^**^	−0.480^**^	1

** *p* < 0.01.

**Table 2:** Comparison between the no/mild c-GVHD group and the moderate/severe c-GVHD group.

Group	*n*	Social constraints	Illness perception	Fear of cancer recurrence	Quality of life
no/mild c-GVHD	196	29.55 ± 5.94	39.00 ± 10.73	13.33 ± 6.22	100.47 ± 21.03
moderate/severe c-GVHD	36	28.93 ± 7.52	38.18 ± 7.44	14.77 ± 4.51	91.40 ± 19.94
*t*		0.551	0.439	1.325	2.487
*P*		0.291	0.661	0.186	0.014

c-GVHD, chronic graft-versus-host disease.

### Tests of mediating effects of illness perceptions and the fear of cancer recurrence

**Figure 2:**
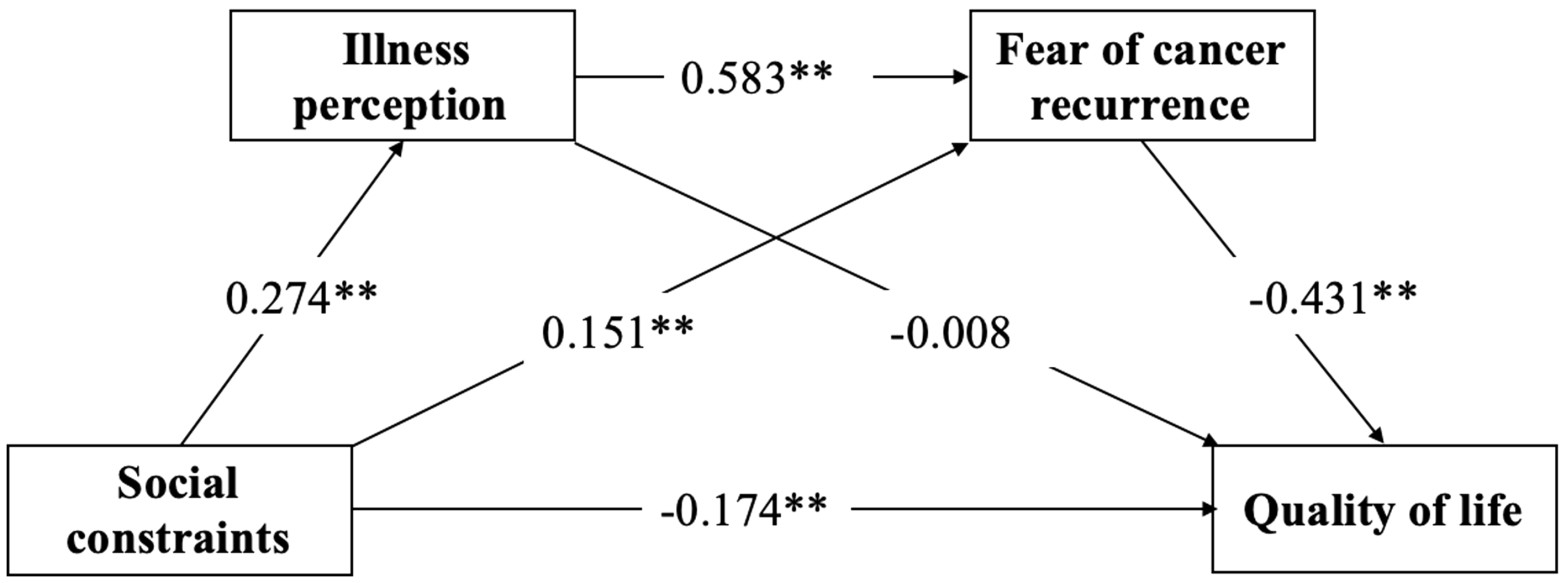
Model of the mediator role of illness perception and fear of cancer recurrence in the relationship between social constraints and quality of life.

Taking social constraints as the independent variable, quality of life as the dependent variable, and illness perceptions and the fear of cancer recurrence as the mediating variables, the test of the chain mediating effect yielded the results shown in Figure 2 and Tables 3, 4. First, the results shown in Table 3 and Figure 2 indicate that social constraints have a significant direct effect on quality of life (β = −0.174, *p* < 0.01), illness perceptions (β = 0.274, *p* < 0.01) and the fear of cancer recurrence (β = 0.151, *p* < 0.01), thus supporting Hypothesis 1.

**Table 3:** The chain mediation model from social constraints to quality of life.

	Illness perception		Fear of cancer recurrence		Quality of life	
	*β*	*t*	*β*	*t*	*β*	*t*
Social constraints	0.274	4.029^**^	0.151	2.670^**^	−0.174	−2.681^**^
Illness perception			0.583	10.299^**^	−0.008	−0.096
Fear of cancer recurrence					−0.431	−5.396^**^
*R* ^2^	0.075		0.411		0.257	
*F*	16.233		69.334		22.863	

** *P* < 0.01.

**Table 4:** Bootstrap mediating effects of social constraints and quality of life.

Paths	Effect	BootSE	95%CI	Relative mediating effect
Total effect	−0.904	0.199	−1.294, −0.513	
Direct effect	−0.503	0.192	−0.890, −0.138	55.64%
Indirect effect	−0.401	0.156	−0.534, −0.124	45.36%
Path a: SC → IP → QoL	−0.006	0.004	−0.026, −0.009	0.66%
Path b: SC → FCR → QoL	−0.192	0.019	−0.092, −0.016	21.24%
Path c: SC → IP → FCR → QoL	−0.203	0.016	−0.088, −0.028	22.46%

SC, social constraints; IP, illness perception; QoL, quality of life; FCR, fear of cancer recurrence.

Second, the results of the analysis of the chain mediating effect showed that illness perceptions and the fear of cancer recurrence have continuous mediating effects on the relationship between social constraints and the quality of life of HCT survivors; the effect value is −0.503, and the proportion of the total effect accounted for by this mediating effect is 45.36%. Specifically, the chain mediating effect is composed of the indirect effects produced by the following three paths. Path a: Social constraints → Illness perception → Quality of life (the effect value is −0.006), which accounts for 0.66% of the total effect. Path b: Social constraints → Fear of cancer recurrence → Quality of life (the effect value is −0.192), which accounts for 21.24% of the total effect. Path c: Social constraints → Illness perception → Fear of cancer recurrence → Quality of life (the effect value is −0.203), which accounts for 22.46% of the total effect. According to Table 4, the 95% confidence intervals (95% CI) of the three mediating pathways did not include 0, indicating that the mediating effects were significant. In other words, Hypothesis 2–4 was supported.

## Discussion

This study principally investigated the chain mediating path of social constraints and quality of life in HCT survivors. Although this study proved that the score of quality of life in the moderate/severe c-GVHD group was significantly lower than that in the no/mild c-GVHD group, the two groups were combined for the mediating effects analysis because of the small sample size in the moderate/severe c-GVHD group. The results showed that social constraints lowers quality of life through the indirect paths of illness perceptions, the fear of cancer recurrence, and the chain mediating path of illness perceptions and the fear of cancer recurrence.

The results of this study showed that social constraints had a negative predictive effect on HCT survivors’ quality of life (H1), as HCT survivors with higher social constraints exhibited worse quality of life. These results were consistent with the findings of several previous studies investigating breast cancer patients (Cui et al., 2021; Rivera-Rivera et al., 2021) and lung cancer patients (Hyland et al., 2019). Under the influence of negative social functioning, HCT survivors are limited in terms of their ability to perceive and seek the social support and emotional expression they need. Survivors who exhibited negative perceptions of their surroundings were more likely to view a cancer diagnosis and HCT as more threatening, more difficult to manage and more stressful, leading to adverse outcomes and reduced quality of life. Therefore, HCT survivors should be provided with social network support to encourage their emotional expression. In the study conducted by Rini et al. (2014), encouraging HCT survivors with moderate-to-severe survivorship problems to participate in an expressive helping intervention was shown to reduce their stress and improve their physical symptoms and quality of life. Another randomized controlled study showed that the provision of continuous nutrition and exercise counseling by professionals could improve the dietary intake and quality of life of patients receiving autologous HCT (Hung et al., 2014). Thus, health professionals should educate survivors concerning cancer self-management and provide them with professional information as well as practical, interpersonal and emotional support.

The results showed that illness perceptions partially mediated the relationship between social constraints and quality of life in HCT survivors (H2). HCT survivors who faced higher social constraints exhibited stronger negative illness perception, were more likely to use passive coping strategies and had lower quality of life. The results of a mixed study showed that although patients feel that the experiences associated with HCT cause their family lives to become more connected and positive, they also fear being perceived as “weak” or “pathetic” by family members and friends (Poloméni et al., 2016). When HCT survivors want to express their feelings regarding the disease or physical discomfort to others, but their relatives and friends exhibit responses of avoidance, obstruction or restriction such as saying “Do not worry. You will be fine,” this situation inhibits the survivors’ desire to engage in further expression and encourages the survivors to adopt negative beliefs, such as the beliefs that they are unable to understand and master the disease, that cancer cannot be cured, and that the consequences of the disease are serious, thus negatively affecting their quality of life.

The results of this study also showed that fear of cancer recurrence played a partially mediating role between social constraints and quality of life (H3), which was similar to the conclusions of the study conducted by Soriano et al. (2018) with respect to breast cancer patients. Recurrence after HCT can entail more difficult treatment, higher medical costs, and even death. As an uncertain but high incidence event, cancer recurrence is a continuously stressful event for survivors themselves as well as their social networks. Fear of cancer recurrence is considered to be a normal response to cancer diagnosis (Lebel et al., 2016). However, when HCT survivors’ desire for self-disclosure of the fear of recurrence was suppressed by social networks, the survivors experienced a sense of disease burden, stigma and uncertainty were aggravated, and their fear of the consequences of cancer recurrence and the burden on their families increased, leading to the development of dysfunctions such as difficulty sleeping, altered health behaviors, and reduced quality of life (Mehnert et al., 2013; Séguin Leclair et al., 2019).

Moreover, we found for the first time that illness perceptions and the fear of cancer recurrence had a chain mediating effect on the relationship between social constraints and quality of life in HCT survivors (H4). This finding means that social constraints can enhance negative illness perceptions of HCT survivors, and illness perceptions can promote their fear of cancer recurrence to further decrease the quality of life. Simonelli et al. (2017) noted that triggers, social factors, cognitive processes, the fear of cancer recurrence and psychological and behavioral outcomes are key components of the theory of fear of cancer recurrence. The cognitive process by which patients experience internal (e.g., physical symptoms and side effects) or external (e.g., cancer-related media, medical follow-up, etc.) trigger cues is affected by factors related to the social environmental, and negative social constraints increase patients’ negative perceptions of disease and subsequently their fear of cancer recurrence. This process establishes a vicious cycle of avoidance, negativity and fear, thus leading to psychological and behavioral dysregulation and ultimately reducing the survivor’s quality of life. Therefore, these results provide new evidence related to the theory of the fear of cancer recurrence in the context of HCT survivors. Furthermore, we find that, among all the paths in which social constraints affects the quality of life of HCT survivors in China, the path of social constraints → illness perceptions → fear of cancer recurrence → quality of life has the highest indirect effect value, accounting for 22.46% of the total effect. This result shows that illness perceptions and fear of cancer recurrence are important for HCT survivors in China whose physical and mental health is severely affected by disease and treatment, and they are also potential important intervention factors in promoting the quality of life of HCT survivors.

### Limitations

This study faced certain limitations. First, we included only HCT survivors in a single hospital in Changsha, Hunan Province, China; thus, the sample size was small. Second, all variables used in this cross-sectional study were collected *via* a self-reported survey; so, we were unable to identify continuous changes in social constraints, illness perceptions, the fear of cancer recurrence, and quality of life. Continuous follow-up investigations should be conducted to investigate HCT survivors. Finally, the research results made it difficult to eliminate bias resulting from demographic and disease-related factors that affect the quality of life of HCT survivors, although strict inclusion criteria were implemented.

## Conclusion

This study shows that social constraints have a negative effect on quality of life in HCT survivors and that illness perceptions and the fear of cancer recurrence have a chain mediating effect on the relationship between social constraints and quality of life. The chain mediating effect of illness perceptions and the fear of cancer recurrence on quality of life indicates that these two variables have important practical significance with respect to improving HCT survivors’ physical and mental health. The study thus serves as a reference for health workers to improve HCT survivors’ quality of life in the future.

## Data availability statement

The raw data supporting the conclusions of this article will be made available by the authors, without undue reservation.

## Ethics statement

The studies involving human participants were reviewed and approved by the Ethics Committee of the Third Xiangya Hospital of Central South University (I 20033). The participants provided their written or verbal informed consent to participate in this study.

## Author contributions

CR was in charge of this whole project and designed and instructed the research. ZS made contributions to data analysis and drafted the manuscript. CL contributed to collecting data. SS instructed the data collection and data analysis. All authors contributed to the article and approved the submitted version.

## Funding

This work was supported by the General project of Hunan Health Commission (B202314018897).

## Conflict of interest

The authors declare that the research was conducted in the absence of any commercial or financial relationships that could be construed as a potential conflict of interest.

## Publisher’s note

All claims expressed in this article are solely those of the authors and do not necessarily represent those of their affiliated organizations, or those of the publisher, the editors and the reviewers. Any product that may be evaluated in this article, or claim that may be made by its manufacturer, is not guaranteed or endorsed by the publisher.
